# Association of lipoprotein(a) with aortic dissection 

**DOI:** 10.1002/clc.23834

**Published:** 2022-08-04

**Authors:** Yiheng Yang, Yuting Hong, Weihua Yang, Zhenzhong Zheng

**Affiliations:** ^1^ Department of Cardiovascular Medicine The First Affiliated Hospital of Nanchang University Nanchang Jiangxi China; ^2^ Department of Renal Medicine The First Affiliated Hospital of Nanchang University Nanchang Jiangxi China; ^3^ Department of Cardiovascular Medicine Affiliated Renhe Hospital of China Three Gorges University Yichang Hubei China

**Keywords:** aortic dissection, case‐control study, lipoprotein(a) [Lp(a)]

## Abstract

**Background:**

Lipoprotein(a) [Lp(a)] is associated with coronary atherosclerotic heart disease, aortic stenosis, stroke, and heart failure. We aimed to determine the relationship between Lp(a) and aortic dissection (AD).

**Methods:**

Two hundred patients with AD were included in our case group. The control group consisted of 200 non‐AD people who were age‐ (±5 years) and gender‐matched to the case group. Data were collected retrospectively, including hypertension, smoking, coronary artery disease, diabetes mellitus, Lp(a), total cholesterol, triglyceride, low‐density lipoprotein cholesterol, and high‐density lipoprotein cholesterol. The association between Lp(a) and AD was studied using univariate and multivariate logistic regression analysis.

**Results:**

Patients with AD had greater median Lp(a) concentrations than non‐AD people (152.50 vs. 81.75 mg/L). Lp(a) was associated with AD in a multivariate logistic regression analysis (odds ratio, 8.03; 95% confidence interval, 2.85–22.62), comparing those with Lp(a) quartile 4 with those with Lp(a) quartile 1. Stratified analysis showed that this relationship was observed in both men and women, as well as in older and younger individuals.

**Conclusions:**

High levels of Lp(a) are strongly associated with AD, independent of other cardiovascular risk factors.

## 1 INTRODUCTION

Aortic dissection (AD) is caused by tearing of the intima of the aorta, exposing the middle layer to pulsatile blood flow. It is a disease with rapid onset and high mortality. Although great breakthroughs have been made in the diagnosis and treatment of AD in recent decades, the mortality rate of AD is still high. It accounts for a large proportion of aortic related deaths.^1^ Every year, the incidence of AD ranges from 3 to 4 per 100 000 persons.^2^ Aging, aneurysm, hypertension, inflammatory diseases, and smoking are known risk factors for AD. In order to better prevent the development of AD and reduce the risk of AD, it is necessary to further explore possible risk factors for the disease.

Lipoprotein(a) [Lp(a)] is a complex particle that is made up of a low‐density lipoprotein (LDL)‐like moiety with an apolipoprotein(a) [apo(a)].^3^ Polymorphisms in the LPA gene, which codes for apo(a), mainly determine the plasma levels of Lp(a).^4^ Plasma Lp(a) levels are less influenced by diet and environment.^5^ In the last 10 years, several large studies have found that Lp(a) is associated with an increased incidence of coronary heart disease, stroke and aortic stenosis; the association was shown to be independent of other risk factors.^6^ At the cellular and molecular level, inflammation and extracellular matrix degradation lead to changes in the structure of the aortic wall, and these changes promote the formation of AD.^7^ According to one study, Lp(a) plays a role in promoting monocyte trafficking to the arterial wall, and its oxidized phospholipid content is important in promoting the inflammatory response of the arterial wall.^8^ A slight increase in Lp(a) level can alter the contents of T‐lymphocyte subpopulations, raising the risk of coronary vascular injury.^9^, ^10^ Therefore, we suggest that high levels of Lp(a)‐mediated inflammatory response and the subsequent vascular injury may be associated with AD.

Previous studies have investigated the possible association of Lp(a) with the risk of AD. However, the results are contradictory. It is uncertain whether elevated Lp(a) levels are associated with the occurrence of AD. Therefore, we aimed to examine the relationship between Lp(a) and AD.

## 2 METHODS

### 2.1 Study patients and design

Patients with AD and age‐ and gender‐matched controls were included in this retrospective study. The research was approved by the Ethics Committee of the First Affiliated Hospital of Nanchang University. General and hospitalization information of patients were extracted from the electronic medical record system at the First Affiliated Hospital of Nanchang University. We included patients diagnosed with AD at the cardiac surgery department between September 1, 2012 and July 31, 2021. AD patients must meet the diagnostic criteria for AD as defined by ACC/AHA.^1^ The exclusion criteria were Marfan's syndrome, aortic ulcer, traumatic AD, patients without Lp(a) assay, and medical records of kidney‐related diseases. People who underwent a health check‐up at the First Affiliated Hospital of Nanchang University were selected as controls.

### 2.2 Definitions

The diagnosis of diabetes mellitus (DM) met the diagnostic criteria of the World Health Organization. Hypertension was defined as having repeated measurements of systolic blood pressure ≥140mmHg or diastolic blood pressure ≥90mmHg, being on antihypertensive medication, or the presence of a diagnosis of hypertension in the clinical information system. The presence of a history of coronary artery disease (CAD) was defined as CAD. Cerebrovascular disease (CVD) was defined as ischemic stroke and hemorrhagic stroke. The diagnosis of dyslipidemia was based on the diagnostic criteria of Azadeh Beheshtian's study.^11^ Smoking referred to both current and former smokers.

### 2.3 Laboratory measurements

Venous blood was collected after an overnight fast for healthy individuals and during hospitalization for AD patients. Serum Lp(a) concentration was determined by latex‐enhanced immunoturbidimetric assay. Total cholesterol (TC) was determined by CHOD‐PAP method. Triglyceride (TG) was determined by GPO‐PAP method. The direcet methd was used for LDL cholesterol (LDL‐C) and high‐density lipoprotein cholesterol (HDL‐C). These serum lipids were measured using a chemistry autoanalyzer (Beckman Coulter).

### 2.4 Statistical analysis

All statistical analyses were carried out using SPSS software (version 25.0 for Windows, SPSS, Inc, Chicago, lllinois). Continuous variables with a normal distribution were presented as the mean ± standard deviation (*SD*). As for skewed distribution variables, they were expressed as median (interquartile range). Frequency (percentage) was used to describe the categorical variables. *T* test was used to compare normally distributed continuous variables and the Mann–Whitney *U* test was used to compare non‐normally distributed continuous variables. Dichotomous variables were compared by using *χ* ^2^ test. We converted Lp(a) as a continuous variable into a categorical variable according to quartiles. The association between Lp(a) and AD was investigated using univariate and multifactorial logistic regression. Furthermore, we included variables in the model that were meaningful from a professional point of view. We also performed a stratified analysis for age and gender. A *p* < .05 was regarded as statistically significant.

## 3 RESULTS

### 3.1 Characteristics of study groups

In this study, the case group contained 200 AD patients. The control group contained 200 age‐ and sex‐matched healthy individuals. As shown in Table 1，the mean age was 53.83 ± 12.10 years old in AD patients and 52.74 ± 11.71 years old in the control group. The median Lp(a) concentration in AD patients was significantly higher than that in the control group (152.50 vs. 81.75 mg/L). The percentages of smoking, hypertension, and CVD were higher in the case group than in the control group, with statistically significant differences between the two groups. AD patients had lower median TG, TC, HDL‐C, and LDL‐C levels than the control group. There were no significant differences in age, sex, CAD, dyslipidemia, or DM between the two groups.

**Table 1:** Characteristics of participants

Characteristics		Cases (*n* = 200)	Controls (*n* = 200)	All (*n* = 400)
Age, years		53.83 ± 12.10	52.74 ± 11.71	53.28 ± 11.91
Male, %		76.00	76.00	76.00
Lp(a), mg/L		152.50 (70.25, 331.50)	81.75 (55.90, 130.08)	99.35 (60.33, 199.00)
TG, mmol/L		1.15 (0.79, 1.72)	1.53 (1.14, 2.15)	1.35 (0.94, 1.97)
TC, mmol/L		4.14 (3.68, 4.73)	4.72 (4.11, 5.39)	4.44 (3.84, 5.13)
HDL‐C, mmol/L		1.18 (0.92, 1.46)	1.21 (1.05, 1.46)	1.20 (1.01, 1.46)
LDL‐C, mmol/L		2.44 (1.93, 3.01)	2.73 (2.33, 3.36)	2.61 (2.11, 3.16)
Dyslipidemia, %		53.50	50.50	52.00
Hypertension, %		77.50	21.00	49.25
CAD, %		2	0.5	1.25
CVD, %		5	0.5	2.75
DM, %		4	3	3.50
Smoking, %		32.5	5	18.75

Abbreviations: DM, diabetes mellitus; HDL, high‐density lipoprotein; LDL, low‐density lipoprotein; TC, total cholesterol; TG, triglyceride.

### 3.2 Lipoprotein(a) and risk of AD

**Figure 1:**
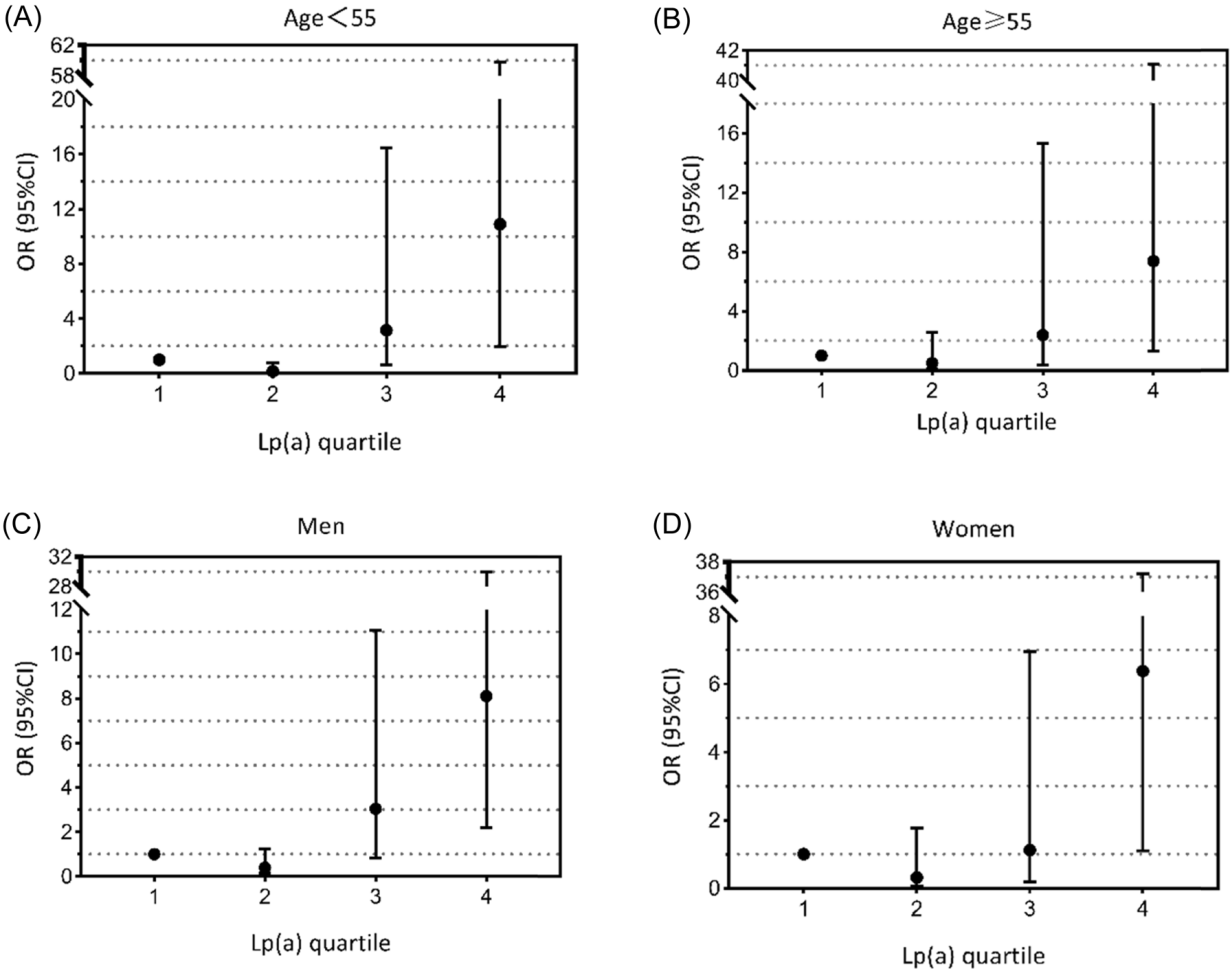
All individuals were stratified by age and gender. The model includes hypertension and smoking

With Lp(a) quartile 1 as the reference, Lp(a) quartile 4 was positively associated with AD in univariate analysis (odds ratio [OR] = 5.18, 95% confidence interval [CI]: 2.65–10.15, *p* < .001). However, Lp(a) quartiles 2–3 did not have such an association. When the variables were adjusted for hypertension and smoking, Lp(a) quartile 4 remained strongly associated with AD (OR = 8.03, 95% CI: 2.85–22.62, *p* < .001). On the basis of model 1, we added CAD, CVD, and DM to the logistic regression analysis. This association of Lp(a) quartile 4 with AD persisted (OR = 7.75, 95% CI: 2.61–23.03, *p* < .001) (Table 2). In addition, all people were stratified by age and sex. After adjusting for hypertension and smoking, the association of Lp(a) with AD was observed in both men and women, as well as in older and younger individuals (Figure 1).

**Table 2:** Association between lipoprotein(a) concentration and aortic dissection

Lp(a) categories	Unadjusted model		Model 1		Model 2	
	OR (95% CI)	*p* value	OR (95% CI)	*p* value	OR (95% CI)	*p* value
Quartile 1	1		1		1	
Quartile 2	0.62 (0.34,1.12)	0.11	0.53 (0.22,1.26)	0.15	0.49 (0.20,1.18)	0.11
Quartile 3	1.14 (0.62,2.09)	0.67	1.98 (0.71,5.47)	0.19	1.99 (0.69,5.73)	0.20
Quartile 4	5.18 (2.65,10.15)	<0.001	8.03 (2.85,22.62)	<0.001	7.75 (2.61,23.03)	<0.001

*Note*: Model 1 includes hypertension and smoking. Model 2 includes hypertension, smoking, CAD, diabetes, and CVD.

Abbreviations: CI, confidence interval; OR, odds ratio.

## 4 DISCUSSION

Our findings clarified the association between Lp(a) concentration and the risk of AD.High levels of Lp(a) are strongly associated with AD, independent of other cardiovascular risk factors. To date, several case‐control studies have investigated the relationship between Lp(a) and AD. In one study, Lp(a) concentrations in AD patients were not significantly different from those in healthy controls.^12^ In another study, serum Lp(a) levels were significantly higher in AD patients than in healthy subjects, and Lp(a) was independently associated with AD in nonsmokers.^13^ The results of our study are similar to those of Xiao‐feng Chen et al,^13^ but different from those of Martin Schillinger et al.^12^ This might be because the sample sizes of our study and the other two studies were small. The sample sizes of the other two studies were both less than 200. A prospective study showed higher Lp(a) levels in blacks than in whites, suggesting that Lp(a) levels differ among races.^14^ Lp(a)‐associated cardiovascular risk may vary by race/ethnicity.^15^, ^16^, ^17^ Therefore, the possible reason for the similarity between our results and those of Xiao‐feng Chen^13^ is that both study subjects were from China. Both renal disease and Lp(a) levels are associated with increased cardiovascular risk, and renal function can influence plasma Lp(a) levels.^18^ Thus, the relationship between renal disease, Lp(a), and cardiovascular risk is complex. In our study, people with medical records of kidney‐related diseases were excluded.

It is known that smoking, hypertension, congenital diseases and inflammatory diseases can lead to the development of AD, while the role of atherosclerosis in the development of AD is unknown.^7^ The unique structure of Lp(a) enables it to promote the occurrence of atherosclerosis, which can precipitate cardiovascular events.^19^ Based on this role of Lp(a) and the findings of our study, we speculate that atherosclerosis may be associated with the development of AD. In order to reduce the risk of Lp(a)‐associated cardiovascular events, several drugs have been shown to reduce Lp(a) levels, such as PCSK9 inhibitors.^20^ LDL‐C also has pro‐atherogenic effect. However, our study showed that the median LDL‐C levels were slightly higher in the control group. Whether atherosclerosis is involved in the development of AD needs further study.

No studies have explored the correlation between DeBakey classification of AD and Lp(a). In Martin Schillinger's study, they only analyzed patients with abdominal aortic aneurysm and thoracic aortic aneurysm with AD, respectively.^12^ Exploring the relationship between Lp(a) and AD according to DeBakey classification may lead to new findings, which need to be further investigated.

Our research has some limitations. Due to the limited evidence provided by the present study, future large cohort studies or Mendelian randomization studies are needed to determine the causal relationship between Lp(a) and AD. Some variables were not included in our study, such as body mass index and homocysteine. This was mainly due to incomplete data for some individuals. Because serum Lp(a) concentrations are influenced by race, studies of Lp(a) and AD among different races are also needed. Due to the complexity of the disease, not all AD patients measured Lp(a) concentrations after overnight fasting.

This study explored the association between Lp(a) concentration and AD. Due to the rapid onset of AD, its high mortality and poor prognosis, AD can lead to serious consequences in most cases. Thus, it is necessary to identify more potential risk factors and intervene on AD risk factors at an early stage to reduce the risk of AD.

## CONFLICTS OF INTEREST

The authors declare no conflicts of interest.

## Data Availability

The data used in this study can be obtained from the corresponding author upon reasonable request.
